# Concentration, Life Stage, Feeding, Density, Flow, and Strain Effects on Formalin Sensitivity in Rainbow Trout (*Oncorhynchus mykiss*)

**DOI:** 10.3390/ani13152425

**Published:** 2023-07-27

**Authors:** Eric R. Fetherman, Brad Neuschwanger, Chris Praamsma, Tracy Davis

**Affiliations:** 1 Aquatic Wildlife Research Section, Colorado Parks and Wildlife, 317 West Prospect Road, Fort Collins, CO 80526, USA; 2 Bellvue Fish Research Hatchery, Colorado Parks and Wildlife, 5500 West County Road 50C, Bellvue, CO 80512, USA; brad.neuschwanger@state.co.us (B.N.);; 3 Bellvue-Watson Fish Hatchery, Colorado Parks and Wildlife, 4936 West County Road 52E, Bellvue, CO 80512, USA

**Keywords:** formalin, rainbow trout, *Oncorhynchus mykiss*, strain, egg, fingerling, aquaculture

## Abstract

**Simple Summary:**

Chemical compounds such as formalin are commonly used to prevent fungal infections on fish eggs and treat external parasites on hatchery-reared fish. However, exposure to formalin can cause mortality, depending on the concentration used and the rearing conditions under which the fish are being treated. Strains within a species can also differ in their sensitivity to formalin. Four experiments were conducted to evaluate egg and fingerling mortality of four strains of rainbow trout (*Oncorhynchus mykiss*) exposed to a range of formalin concentrations. How mortality was affected by the conditions under which rainbow trout were being treated was also examined. Strains exhibited differential sensitivity to formalin in both the egg and fingerling life stages. Hatchery rearing conditions were also found to differentially affect mortality, both within and across strains. Therefore, formalin concentration, rearing conditions, and potential strain differences in sensitivity should be considered prior to initiating large-scale formalin treatments.

**Abstract:**

Formalin is one of the most widely used and effective chemotherapeutic compounds for treatment of fungal infections and external parasites of fish eggs and fish. However, exposure to formalin can cause mortality in eggs and fingerlings, dependent upon the concentration used and the rearing conditions in which fish are treated. Additionally, strains within a species can exhibit differential susceptibility to formalin. Four experiments were conducted to evaluate the differential sensitivity to formalin of four rainbow trout (*Oncorhynchus mykiss*) strains in both the egg and fingerling life stages. Eggs were exposed to concentrations of 1667, 2000, and 5000 ppm formalin, and sensitivity differed among the strains when formalin concentration exceeded 2000 ppm. Exposure to higher formalin concentrations (i.e., 5000 ppm) as eggs did not increase mortality when fish were re-exposed to concentrations of 0, 167, 250, or 500 ppm formalin at 77 mm total length (TL). Fish size affected formalin sensitivity, with larger fish (128 mm TL) exhibiting higher rates of mortality than fish ≤ 77 mm TL when exposed to 250 ppm formalin. The effects of crowding, feeding, flow, and density on the formalin sensitivity of 77 mm TL fish were also investigated. Mortality increased in fish crowded away from the inflow to prevent contact with formalin as it entered the tank, potentially the result of an increase in density index within the crowded tanks. Feeding fish on the day they were treated caused mortality to increase by 5.4 to 8.8% in fish exposed to 167 and 250 ppm formalin, respectively, and mortality differed by strain. Reducing flows by half resulted in doubled to quadrupled mortality, and increased densities resulted in increased mortality in some strains but not others. Hatchery managers should consider what effect rearing conditions and formalin concentrations might have on the strain of fish being treated, prior to large-scale treatment.

## 1. Introduction

Formalin is one of the most effective and widely used compounds in fish culture for therapeutic treatment of fungal infections and external parasites of fish eggs and fish [1,2]. Species of the fungal order Saprolegniales and other aquatic fungi are ubiquitous in the water supply of fish hatcheries and often cause serious disease problems, especially in intensive culture and high-density systems [3]. Formalin has been shown to reduce fungal growth on fish eggs and decrease egg mortality [4]. Concentrations as low as 250 ppm can effectively prevent fungal infections on rainbow trout (*Oncorhynchus mykiss*) eggs [3,5]. However, Marking et al. [3] found that at 1000 ppm, formalin not only prevented infection, but also decreased existing infection and increased hatching rates at exposure times ranging from 15 to 60 min. In addition to being a fungicide, formalin has been shown to be an egg disinfectant, reducing bacterial abundance on the surface of the egg at concentrations of up to 2000 ppm [6].

Formalin has become one of the most-used chemotherapeutic agents for the control and treatment of diseases in hatcheries because of its versatility and effectiveness, particularly for controlling and treating diseases of the skin, fins, and gills [7]. For treating fish, formalin is effective against most ectoparasites, including *Trichodina*, *Costia*, *Ichthyophthirius*, and monogenetic trematodes [8]. The effective dose of formalin used to treat fish is determined by the time that the fish are subjected to the treatment and the tolerance of the animals [7]. Typical formalin exposure concentrations range from 125 to 250 ppm for up to one hour [8]. However, concentrations of up to 400 ppm have been used experimentally in toxicity tests [9,10]. A poll of Colorado Parks and Wildlife (CPW) hatchery managers found that a range of concentrations from 130 to 250 ppm were used, with the most common treatment being 167 ppm formalin for 30 min. A similar poll conducted by Piper and Smith [11] showed the most-frequently used formalin treatments by Federal and State fishery personnel throughout the United States were 167 to 250 ppm for 1 h.

Multiple formalin treatments are often needed when water temperatures are higher, formalin concentrations are lower, or infestations of external parasites are more severe [8]. Although some studies have shown little effect of multiple treatments to the gills [12], fish growth and appetite, and even improvements in fin condition and corneal opacity [13], others have shown damaging effects of a single exposure, let alone multiple exposures, to formalin. In rainbow trout, exposure to formalin has been shown to cause damage to the gills [14,15,16,17], liver [16,18], and spleen [14]. Severe pathological changes observed in the gills of formalin-treated trout resulted in dysfunction of the gill epithelium, making the fish unable to osmoregulate [14]. Additionally, treatments with formalin may damage the fish epidermis, leaving the fish susceptible to other pathogens [19]. Therefore, despite the benefits of formalin use for the reduction of parasites in cultured fish populations, there may be long-term effects or mortality associated with exposure beyond the mortality associated with the parasites for which the fish are being treated.

Differential sensitivity (measured by mortality) to formalin has been demonstrated for various strains of rainbow trout when exposed post hatch [11]. However, there has been little research on differential strain sensitivity to formalin exposure during egg incubation. The goal of the experiments described herein was to evaluate if there was differential sensitivity to formalin of four rainbow trout strains in both the egg and fingerling life stages, and determine what hatchery rearing conditions may affect differential mortality within the fingerling life stage. To that end, four experiments were conducted. The objective of the first experiment was to evaluate the effect of formalin concentration on rainbow trout egg and post-hatch mortality. The second experiment was designed to determine if prior exposure to higher formalin concentrations as eggs affected formalin sensitivity of rainbow trout fingerlings. The objective of the third experiment was to examine the effects of fish size, as well as rearing conditions, including crowding fish down, feeding, flow, and density, on the formalin sensitivity of fingerling rainbow trout. The fourth experiment similarly evaluated effects of density, as well as whether multiple formalin treatments increased rainbow trout fingerling mortality. The results from these experiments can be used to determine the potential effects of concentration, rearing conditions, and strain sensitivity differences when using formalin to treat rainbow trout eggs and fingerlings.

## 2. Materials and Methods

### 2.1. Site Description and Fish Production

Formalin experiments were conducted at the CPW Bellvue Fish Research Hatchery (BFRH; Bellvue, CO, USA). The BFRH is supplied with water from an on-site well that maintains a temperature of 12 °C, a dissolved oxygen concentration of 9 mg/L, a pH of 7.0, and a water hardness of 120 mg/L as CaCO_3_ year round. The well has no surface connection or known biota, and water in both the hatchery and isolation buildings where these experiments were conducted is first use, i.e., water is not subject to serial reuse and has not passed through any other groups of fish prior to use.

*Myxobolus cerebralis*, the parasite responsible for salmonid whirling disease, caused a severe decline in wild rainbow trout populations following its establishment in coldwater systems throughout Colorado [20,21,22]. Current management efforts are focused on stocking rainbow trout that are genetically resistant to the parasite [23]. However, *M. cerebralis*-resistant rainbow trout are a relatively new addition to the state aquaculture system [24]. Therefore, four *M. cerebralis*-resistant rainbow trout strains were evaluated for their formalin sensitivity in these experiments. The German Rainbow (GR) is a domesticated, hatchery-derived strain that was exposed to *M. cerebralis* over many generations in Germany and is more resistant to *M. cerebralis* than many other rainbow trout strains found in North America [25]. The Harrison Lake rainbow trout (HL; origin: Harrison Lake, MT, USA [26]) is one of the wild strains crossed with the GR to create a fish capable of surviving and reproducing in the wild [27,28]. Brood stocks of GR and HL fish are maintained at the CPW BFRH. A first-generation cross of the GR and HL strains (hereafter the GR × HL 50:50 strain) and a second-generation backcross of the GR × HL 50:50 and the GR (hereafter the GR × HL 75:25 strain) were also included in these experiments. These strains, previously evaluated for their critical dissolved oxygen tolerances [24], are maintained as brood stock in Colorado’s state hatchery system, and produced and stocked for recreational purposes statewide.

Spawning for all four experiments described herein occurred in the December prior to each experiment, and we followed the annual spawning procedures used by the BFRH to create their replacement brood fish for each strain. GR eggs were obtained by spawning pairs of two-year-old GR females with three-year-old GR males. HL eggs originated from pairs of two-year-old HL females spawned with three-year-old HL males, as well as pairs of three-year-old HL females spawned with two-year-old HL males, dependent upon availability of brood sexes and age classes. The GR × HL 50:50 eggs were similarly obtained from pooled pairs of two- or three-year-old GR females spawned with two- or three-year-old HL males. Two-year-old GR × HL 50:50 males or females were spawned with either two- or three-year-old GR males or females to obtain GR × HL 75:25 eggs (Table 1). Spawning and egg water hardening occurred alongside the outdoor raceways in which the brood fish were held. After fertilization, eggs from each spawning pair were pooled into coolers by strain (four in total) and water hardened in a 50 ppm iodine solution for one hour. Before entering the hatchery, eggs were submerged in a 100 ppm iodine bath for an additional ten minutes to prevent transporting bacteria or other pathogens into the hatchery building.

Egg cups placed within vertical Heath stacks served as the strain replicates for assessing mortality within and across formalin concentrations in each of the four experiments. Although the number of replicate egg cups and formalin concentrations to which the eggs were exposed differed among experiments (see details for each experiment below), the following procedure was used to distribute eggs into egg cups prior to the start of each experiment. Eggs from each strain were initially counted out by hand into a measuring cup to determine the number of ounces that contained 500 eggs of that specific strain (conducted once per strain per year). Using this initial measurement, eggs were measured out instead of counted out, to distribute approximately 500 eggs into each 7.6 cm diameter, screen-bottomed PVC egg cup contained within the Heath stack trays. Eggs differed in size across strains and years. However, final egg numbers (±SD) using this technique averaged 507 (±30) eggs per egg cup, with no differences in the average number of eggs per cup across years or among the strains.

### 2.2. Experiment 1: Egg Formalin Sensitivity

Three formalin concentrations, 1667, 2000, and 5000 ppm, were used to determine the effect of concentration on rainbow trout egg and post-hatch mortality. Note that although concentrations of up to 2000 ppm comply with the approved label claims for use of formalin as a fungicide for finfish eggs, the concentration of 5000 ppm exceeds allowable concentrations and was used in this experiment for research purposes only. Veterinarian approval and oversight is needed to use higher concentrations than those approved for use on the label. A traditional control of no formalin treatment was not included in this and the other experiments for several reasons. First, previous experiments containing control concentrations of 0 ppm formalin had shown that survival to hatch is ≤42% [3,29]. Although handpicking without formalin treatment can increase survival to hatch to up to 84% [29], it is not a standard practice used in Colorado hatcheries. Second, we wanted to avoid the potential for increased infection of brood replacement eggs that were being reared in the Heath stacks along with the eggs for this experiment. Lastly, we were constrained by the number of tanks available to assess post-hatch mortality while maintaining replication across strains and formalin concentrations, and therefore, the control concentration of 0 ppm was dropped to allow replication within the other three concentrations. A concentration of 1667 ppm was included in the experiment because it was the standard concentration used to treat eggs at the BFRH and many other Colorado hatcheries. An increased concentration of 2000 ppm was chosen because it is used to treat eggs in areas where higher fungal infection rates occur [8]. The final concentration of 5000 ppm was included because it was five times that shown to be effective for fungal control (1000 ppm [3]), and was thought to be toxic to the eggs of at least one of the rainbow trout strains included in the experiment, allowing for assessment of differential sensitivity.

To begin the experiment, approximately 500 eggs from each of the four strains were distributed to 24 egg cups placed in the top four trays within each of three Heath stacks using the measurement procedure described above. Strains were assigned to the egg cups using a random number generator. The number of tanks available for evaluating post-hatch mortality (24 tanks) constrained the number of replicate egg cups per strain that could be included in the Heath stacks, such that each stack contained two replicate egg cups per strain, eight egg cups in total, exposed to a given formalin concentration. Each of the three Heath stacks was treated with one of the three formalin concentrations (Table 2). Treatment with formalin began the day after eggs were placed in the Heath stacks, with treatments occurring every other day until the eggs eyed (approximately 14 days, seven formalin treatments). Dead or unhealthy eggs were not removed during this time, to prevent increased mortality due to disturbance. Formalin (37% formaldehyde by volume) was administered using a 3.8 L chicken feeder with a hole sized to allow the feeder to drain at a rate of 15.2 L per hour, and an exposure period of 15 min in the 18.9 Lpm flow-through Heath stacks. The desired concentrations of 1667, 2000, and 5000 ppm were achieved by adding 473, 568, and 1420 mL of formalin, respectively, to the chicken feeders filled with hatchery water.

Once the eggs eyed, treatments ceased. Eyed eggs were removed from the egg cups and physically shocked by pouring the eggs into a floating tray in a hatchery raceway where the dead and unfertilized eggs were identified, counted, and removed. Eggs were then moved into 24 7.6 L tanks, each containing the eggs from one egg cup to maintain replication, and held until they hatched. Hatching began approximately fourteen days after the eggs were moved to the tanks, and it took an additional seven days for all eggs in a tank to hatch. Pre-hatch mortality was calculated for each replicate of strain and formalin concentration using the total number of eggs removed after being physically shocked and any remaining unhatched eggs at the end of the hatching period. After hatching, sac-fry took seven to 10 days at 12 °C to absorb their yolk sacs and swim up. Deformed and unhealthy fish that did not survive to swim up were removed and counted, and this number was used to calculate post-hatch mortality. Lastly, swim-up fish were counted and added to the number of eggs and deformed fish previously removed, to back-calculate the number of eggs in each replicate at the beginning of the experiment, which was used for all mortality calculations. The sum of pre-hatch and post-hatch mortality was used to calculate total mortality for each strain and formalin concentration (following [30]).

Data for all four experiments presented herein were analyzed using a general linear model (GLM) as implemented in SAS Proc GLM [31]. Mortality data were arcsine square root transformed, prior to analysis. For this experiment, we created three model sets, one each for pre-hatch, post-hatch, and total mortality. The model sets included an intercept-only model, as well as singular, additive, and interactive effects of strain and formalin concentration on mortality. Models were ranked using Akaike Information Criterion corrected for small sample sizes (AIC*_c_*), compared using AIC*_c_* differences (ΔAIC*_c_*), and ranked using model weights (*w_i_*). Parameter estimates were reported from the candidate model(s) with the lowest AIC*_c_* value(s) [32], and we used a lack of overlap in two times the standard error (2SE) around the parameter estimates to determine differences among strains and formalin concentrations.

### 2.3. Experiment 2: Fingerling Formalin Sensitivity

All methods of egg handling, formalin treatment, and hatching and rearing swim-up fry used in the first experiment were similarly used in the second experiment. The primary difference between the two experiments was that only two formalin concentrations were used in the second experiment, 1667 and 5000 ppm. The concentration of 2000 ppm was dropped, based on the results of the first experiment and because a wider range in concentrations used to treat eggs was expected to result in measurable differences in fingerling mortality if prior exposure affected fingerling formalin sensitivity. Using only two concentrations allowed more strain replicates to be included in the experiment, with three replicate egg cups per strain in each Heath stack (Table 3). Strain replication was similarly maintained from egg through swim up in the tanks. Pre-hatch, post-hatch, and total mortality was calculated for each strain and formalin concentration using the same methods, and data were analyzed using the same AIC*_c_* approach, as described for the first experiment.

Upon conclusion of the egg formalin sensitivity portion of the experiment, strain replicates were combined into eight troughs, each containing a strain previously exposed to 1667 or 5000 ppm formalin as eggs (e.g., trough 1: GR exposed to 1667 ppm, trough 2: GR exposed to 5000 ppm, etc.), to grow fish up to the appropriate size for the fingerling formalin sensitivity portion of the experiment. All groups were fed Rangen trout feed (Buhl, Idaho) at 2.5% of their body weight per day (BW/d), and were reared under similar environmental conditions (i.e., flows, temperatures, etc.) until they reached approximately 77 mm total length (TL; fingerlings). Two weeks prior to initiation of the first trial, fish were anesthetized using tricane methanesulfonate (MS-222; Syndel, Ferndale, WA, USA) and marked with a visual implant elastomer (VIE) tag in the adipose tissue behind both eyes, preventing misidentification if a tag was lost from one side during experimentation. Four different colors were used to mark the fish, one for each strain. The formalin concentration to which fish had been exposed as eggs was not included in the marking process, since separate trials were run using fish from each egg formalin concentration.

Four formalin exposure trials were conducted, during which formalin treatments occurred for either 30 or 60 min, with the same treatment duration occurring in all tanks within a given trial. Twelve 74.8 L tanks were used in each trial, providing three replicate tanks treated with one of four randomly assigned formalin concentrations, 0, 167, 250, and 500 ppm (Table 4). Note that the concentration of 500 ppm exceeds allowable concentrations for the use of formalin as a parasiticide for finfish, and was used in this experiment for research purposes only. Formalin treatments were applied to all 12 tanks in the same day. Trials were conducted in the following order: (1) 30 min treatment of fingerlings previously exposed to 1667 ppm as eggs, (2) 60 min treatment of fingerlings previously exposed to 1667 ppm as eggs, (3) 30 min treatment of fingerlings previously exposed to 5000 ppm as eggs, and (4) 60 min treatment of fingerlings previously exposed to 5000 ppm as eggs. Five days prior to the start of a trial, 20 fish of each strain were randomly distributed to each of the 12 tanks, such that each one contained a total of 80 fish per tank (density index [DI] = 0.18; [8]). This provided strain replication with consideration to both the egg formalin concentrations (1667 or 5000 ppm) and the fingerling formalin concentrations (0, 167, 250, and 500 ppm; Table 4). Fish continued to be fed at 2.5% BW/d within the treatment tanks until the day prior to the start of a trial.

Tank flow rates were set to 7.6 Lpm to achieve three or six full turnovers of water during a 30 or 60 min trial, respectively, as well as to standardize formalin delivery rate to achieve the desired concentration. Peristaltic meter pumps were used to deliver the formalin at a rate of 1.26 mL per minute, 1.89 mL per minute, or 3.78 mL per minute for the 167, 250, and 500 ppm concentrations, respectively. Hatchery water was delivered via a peristaltic pump at a rate of 3.78 mL per minute for the control concentration of 0 ppm. The length and weight of mortalities occurring during and after exposure were recorded by strain, identified by VIE color, from each tank.

Formalin is known to remove oxygen from the water at a rate of 1 ppm oxygen for every 5 ppm formalin within 30–36 h [8]. Dissolved oxygen concentrations were therefore monitored at the tank outflow during treatment. However, in this experiment, as well as in experiments three and four described below, oxygen concentration ranged from 6.9 to 8.9 ppm, and never dropped below levels considered optimal for trout (≥5 ppm; [8]). Dissolved oxygen concentration was therefore not included as a factor affecting mortality during analysis.

Fish treated with excessive concentrations of formalin may suffer delayed mortality, with the onset of death typically occurring within 1 to 24 h of treatment, but potentially up to 48 to 72 h later, depending on the size or condition of the fish and the water temperatures [8]. Therefore, fish were retained for five days following formalin exposure, so that residual mortality could be recorded. The date and time at which mortalities were found was recorded, as was the length, weight, and strain. Fish remaining after five days were euthanized using an overdose of MS-222, counted, measured, and weighed. Following the removal of the fish, the tanks were cleaned and prepared for the next trial, until all four trials had been completed.

A GLM implemented in SAS Proc GLM was used to evaluate fingerling mortality data from the four trials. Data from all four trials were combined into one analysis, the model set for which included an intercept-only model, and singular and additive effects of egg formalin concentration (1667 or 5000 ppm), fingerling formalin concentration (0, 167, 250, or 500 ppm), treatment duration (30 or 60 min), and strain (GR, HL, GR × HL 50:50, and GR × HL 75:25) on fingerling mortality. Additionally, the model set included interactive effects of egg formalin concentration and fingerling formalin concentration, testing if previous exposure to higher concentrations of formalin affected fingerling mortality, as well as egg formalin concentration, fingerling formalin concentration, and duration of exposure. Models were ranked using AIC*_c_*, compared using ΔAIC*_c_*, and ranked using *w_i_*. Parameter estimates were reported from the candidate model(s) with the lowest AIC*_c_* value(s), and we used a lack of overlap in 2SE around the parameter estimates to determine differences among strains and formalin concentrations.

### 2.4. Experiment 3: Fish Size, Feeding, Crowding, Flow, and Density

Eggs in the third experiment were only exposed to a concentration of 1667 ppm formalin. A single Heath stack was used for egg incubation, which contained five replicate egg cups per strain (20 total), randomly distributed throughout the trays, to produce enough fish for subsequent fingerling formalin sensitivity trials. Although the same methods were used to evaluate pre-hatch, post-hatch, and total mortality, mortality did not differ in any strain from that observed in the egg formalin concentration of 1667 ppm in the previous two experiments. Therefore, no egg mortality results are presented for this experiment.

Similar to experiment two, after fish had hatched and swum up, strain replicates were combined into four troughs, one per strain, and fish were reared to approximately 38 mm, 77 mm, or 128 mm TL, depending on which trial(s) (described below) the fish were to be used for. All groups were fed Rangen trout feed at 2.5% BW/d and reared under similar environmental conditions. As with experiment two, fish were marked with a different color VIE tag for each strain two weeks prior to the initiation of the first formalin sensitivity trial. Five days prior to a trial, an equal number of fish from each strain were distributed to each of nine or twelve 74.8 L tanks (the number varied by trial), and continued to be fed 2.5% BW/d until the day prior to the trial, with the exception of the feeding trial.

The third experiment consisted of seven separate formalin exposure trials in which fish were exposed to 0, 167, or 250 ppm of formalin for 30 min. Trials one and seven were used to examine the effects of fish size on formalin sensitivity, utilizing 38 mm TL fish for the first trial and 128 mm fish for the seventh trial. In both trials, nine experimental tanks were used, three randomly assigned replicate tanks of each formalin concentration. Flow for all tanks was set to 7.6 Lpm. Tanks contained 20 fish of each strain (80 fish in total), with a DI of 0.06 for the 38 mm fish and a DI of 0.44 for the 128 mm fish. Mortality data for 77 mm TL fish were obtained from tanks in trials two, three, and four, which contained the same number of fish (80) and had the same flow rates as those in trials one and seven, to evaluate differences in formalin sensitivity across the three size classes.

Trials two, three, and four were used to determine the effects of density and flow on strain formalin sensitivity. Four combinations of density and flow were tested: (1) normal density (20 fish per strain, 80 fish total; DI = 0.16) and normal flow (7.6 Lpm), (2) normal density and reduced flow (3.8 Lpm; reduced immediately prior to formalin exposure and increased one hour after), (3) increased density (40 fish per strain, 160 fish total; DI = 0.32) and normal flow, and (4) increased density and reduced flow. The two densities represented typical densities found in Colorado hatcheries. The flows essentially simulated either normal operations or an unintentional reduction in water flow prior to treatment, e.g., due to pipe clogs, equipment failure, or accidental reduction at a valve, with flow issues corrected shortly after treatment. Each trial utilized 12 experimental tanks, and the above combinations of density and flow, as well as formalin concentration, were randomly assigned within and across trials such that three replicates of each density, flow, and formalin concentration were completed by the end of the three trials.

Colorado hatchery managers previously observed that GR strain fish tended to congregate under the water inflow during formalin treatment, which has the potential to expose the GR to formalin hot spots prior to the diffusion of formalin throughout the water column. Trial five was conducted to determine if moving fish away from the inflow could reduce mortality during treatment. To test this, fish were moved away from the inflow into the lower two-thirds of the tank, using a crowding screen, where they remained for the duration of the treatment, allowing formalin to diffuse throughout the water column before contacting the fish. Nine experimental tanks with flows set to 7.6 Lpm were used, with three randomly assigned replicate tanks for each formalin concentration. Each tank contained 20 fish per strain (80 fish in total; DI = 0.16). After treatment concluded, the crowding screen was removed to allow full use of the tank.

Trial six was used to simulate an accidental or unintentional feeding on the day of treatment. Similar to the previous trial, nine experimental tanks with flows set to 7.6 Lpm and containing 20 fish per strain (80 fish in total; DI = 0.16) were used, with three randomly assigned replicate tanks of each formalin concentration. All tanks were fed a normal ration (2.5% BW/d) 30 min prior to formalin exposure. For statistical comparisons, data were obtained from tanks in trials two, three, and four, containing the same number of fish (80) but that had last been fed the day prior to treatment, to examine the effects of feeding on the day of treatment on formalin sensitivity.

Peristaltic meter pumps were used to deliver the formalin at a rate of 1.26 or 1.89 mL per minute for the 167 and 250 ppm concentrations, respectively, in all trials. Hatchery water was delivered via a peristaltic pump at a rate of 1.89 mL per minute for the control concentration of 0 ppm. The length and weight of mortalities occurring during and after exposure were recorded by strain, identified by VIE color, from each tank. Additionally, fish were retained for five days following formalin exposure so that residual mortality could be recorded. The date and time at which mortalities were found was recorded, as was the length, weight, and strain. Fish remaining after five days were euthanized using an overdose of MS-222, counted, measured, and weighed. Following the removal of the fish, the tanks were cleaned and prepared for the next trial, until all seven trials had been completed.

Four separate GLM analyses implemented in SAS Proc GLM were conducted using the data collected across the seven trials. Each of the four model sets included an intercept-only model, as well as singular, additive, and interactive combinations of factors affecting mortality, including strain and formalin concentration (all model sets), fish size (model set one), feeding (model set two), crowding (model set three), and density and flow (model set four). Models were ranked using AIC*_c_*, compared using ΔAIC*_c_*, and ranked using *w_i_*. Parameter estimates were reported from the candidate model(s) with the lowest AIC*_c_* value(s), and we used a lack of overlap in 2SE around the parameter estimates to determine differences among strains and formalin concentrations.

### 2.5. Experiment 4: Density and Multiple Exposures

Eggs in the third experiment were only exposed to a concentration of 1667 ppm formalin. A single Heath stack was used for egg incubation, which contained seven replicate egg cups per strain (twenty-eight in total), randomly distributed throughout the trays, to produce enough fish for subsequent fingerling formalin sensitivity trials. Pre-hatch, post-hatch, and total mortality did not differ in any strain from what was observed in the egg formalin concentration of 1667 ppm in the previous three experiments. Therefore, no egg mortality results are presented for this experiment.

Similar to experiments two and three, after the fish had hatched and swum up, strain replicates were combined into four troughs, one per strain, and the fish were reared to approximately 77 mm TL. All groups were fed Rangen trout feed at 2.5% BW/d and reared under similar environmental conditions. As with the previous two experiments, fish were marked with a different color VIE tag for each strain two weeks prior to the initiation of the first formalin sensitivity trial. Five days prior to a trial, an equal number of fish from each strain were distributed to each of 12 74.8 L tanks (the number varied by trial), and continued to be fed 2.5% BW/d until the day prior to the trial. Fish were also fed on the days between treatments.

Four trials were conducted in which fish were exposed to 0, 167, 250, and 500 ppm of formalin for 30 min on the first, third, and fifth days of the trial, to evaluate the effects of multiple exposures to formalin on mortality. Formalin concentrations were randomly assigned to the tanks such that each trial contained three replicate tanks for each concentration. Densities were the same across all 12 tanks within a trial, but differed among trials. Tanks in the first trial contained 20 fish per strain (80 fish in total; DI = 0.10), tanks in the second trial contained 80 fish per strain (320 fish in total; DI = 0.49), tanks in the third trial contained 40 fish per strain (160 fish in total; DI = 0.30), and tanks in the fourth trial contained 10 fish per strain (40 fish in total; DI = 0.09).

Peristaltic meter pumps were used to deliver the formalin at a rate of 1.26, 1.89, or 3.78 mL per minute for the 167, 250, and 500 ppm concentrations, respectively, in all trials. Hatchery water was delivered via a peristaltic pump at a rate of 3.78 mL per minute for the control concentration of 0 ppm. The length and weight of mortalities occurring during and after exposure were recorded by strain, identified by VIE color, from each tank. Additionally, fish were retained for five days following the third and final formalin exposure, so that residual mortality could be recorded. The date and time at which mortalities were found was recorded, as was the length, weight, and strain. Fish remaining after five days were euthanized using an overdose of MS-222, counted, measured, and weighed. Following the removal of the fish, the tanks were cleaned and prepared for the next trial until all four trials had been completed.

Data from all four trials were combined into a single GLM analysis implemented in SAS Proc GLM. The model set included an intercept-only model, and singular, additive, and interactive combinations of strain, formalin concentration, density, and treatment number (one, two, or three; modeled as a trend). Models were ranked using AIC*_c_*, compared using ΔAIC*_c_*, and ranked using *w_i_*. Parameter estimates were reported from the candidate model(s) with the lowest AIC*_c_* value(s), and we used a lack of overlap in 2SE around the parameter estimates to determine differences among strains and formalin concentrations.

## 3. Results

### 3.1. Experiment 1: Egg Formalin Sensitivity

**Figure 1:**
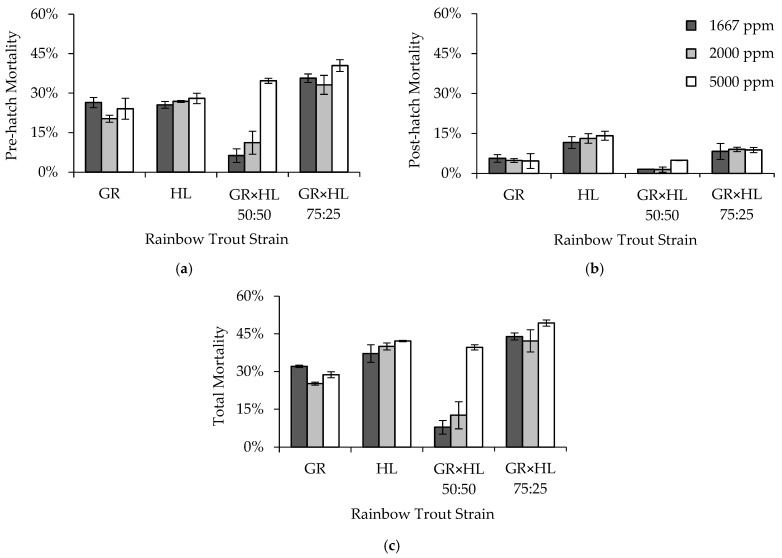
Average (**a**) pre-hatch, (**b**) post-hatch, and (**c**) total mortality (2SE bars) by strain and formalin concentration for rainbow trout (*Oncorhynchus mykiss*) eggs exposed to formalin concentrations of 1667, 2000, and 5000 ppm in Experiment 1. Note that the mortality axes are reduced, to show differences among strains and formalin concentrations.

An interaction between formalin concentration and strain was the best predictor of pre-hatch mortality in Rainbow Trout eggs exposed to 1667, 2000, or 5000 ppm formalin (Table 5). Eggs exposed to 5000 ppm exhibited higher average (±2SE) mortality (31.8 ± 7.3%) across strains than did those exposed to either 1667 or 2000 ppm (23.4 ± 12.3% and 22.9 ± 9.4%, respectively). On average, across formalin concentrations, the GR × HL 50:50 strain exhibited the lowest egg mortality (17.4 ± 17.4%), with increased mortality exhibited by the GR (23.6 ± 3.6%) and HL (26.8 ± 1.4%) strains. The GR × HL 75:25 exhibited the highest egg mortality, both on average (36.4 ± 4.3%) and within each formalin concentration (Figure 1). In addition, the GR × HL 75:25 exhibited a sensitivity to formalin, with an increase in mortality of 4.7–7.3% when exposed to 5000 ppm formalin. However, the greatest sensitivity to formalin was exhibited by the GR × HL 50:50, with a ≥23.5% increase in mortality when eggs were exposed to a formalin concentration of 5000 ppm (Figure 1). Post-hatch mortality differed by strain (Table 5), with the HL strain exhibiting higher post-hatch mortality than the other strains (Figure 1).

Total mortality was best predicted by an interaction between formalin concentration and strain (Table 5). The HL, GR × HL 50:50, and GR × HL 75:25 all showed an increase in mortality between exposure to either 1667 or 2000 ppm and 5000 ppm formalin, with the largest increase in mortality (27%) occurring in the GR × HL 50:50. Unexpectedly, in the GR strain, the highest mortality occurred when eggs were exposed to a formalin concentration of 1667 ppm (Figure 1). On average, the highest total mortality occurred when eggs were exposed to 5000 ppm formalin (39.9 ± 8.5%), relative to concentrations of 1667 and 2000 ppm (30.2 ± 15.7% and 30.0 ± 13.8%, respectively). The GR × HL 75:25 exhibited the highest total mortality of the strains (GR: 28.7 ± 4.0%; HL: 39.8 ± 2.9%; GR × HL 50:50: 20.0 ± 19.8%; GR × HL 75:25: 45.1 ± 4.3%).

### 3.2. Experiment 2: Fingerling Formalin Sensitivity

**Figure 2:**
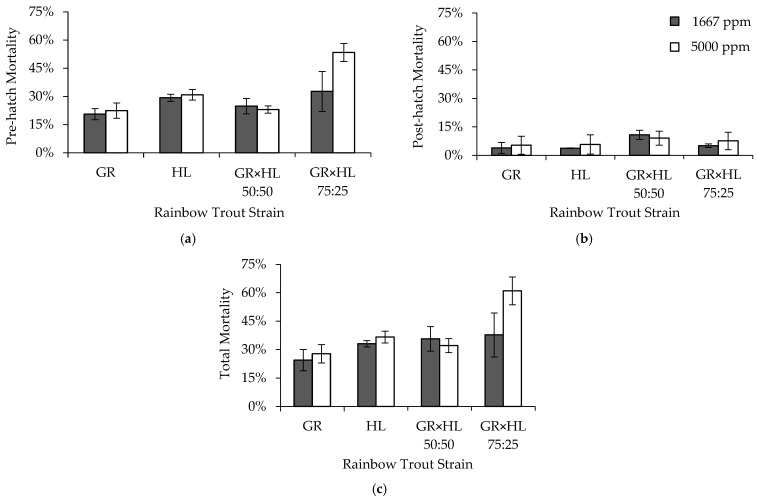
Average (**a**) pre-hatch, (**b**) post-hatch, and (**c**) total mortality (2SE bars) by strain and formalin concentration for rainbow trout (*Oncorhynchus mykiss*) eggs exposed to formalin concentrations of 1667, and 5000 ppm in Experiment 2. Note that the mortality axes are reduced to show differences among strains and formalin concentrations.

Pre-hatch mortality primarily differed by strain, although a formalin concentration by strain interaction also explained some of the variability in pre-hatch mortality (Table 6). The GR × HL 75:25 exhibited the highest egg mortality (43.0 ± 20.7%), with the second highest mortality observed in the HL strain (30.1 ± 1.5%), and lowest mortality observed in the GR strain (21.5 ± 1.9%) and GR × HL 50:50 (23.9 ± 1.8%). On average, mortality was higher when eggs were exposed to 5000 ppm formalin (32.4 ± 14.5%) compared to 1667 ppm formalin (26.8 ± 5.3%). The only strain to exhibit a sensitivity to formalin during the egg life stage was the GR × HL 75:25, with an increase in mortality of 20.8% between 1667 and 5000 ppm formalin (Figure 2). Post-hatch mortality differed by strain (Table 6), with the GR × HL 50:50 exhibiting the highest mortality, but mortality for all strains was ≤ 9.9 ± 1.7%.

Total mortality differed primarily by strain, although additive and interactive effects of formalin concentration also explained some of the variability in total mortality (Table 6). The GR strain exhibited the lowest, and the GR × HL 75:25 the highest, total mortality (26.1 ± 3.3% and 49.3 ± 23.2%, respectively). Mortality was 6.6% higher on average when eggs were exposed to a formalin concentration of 5000 vs. 1667 ppm, and the difference between the concentrations was largely driven by the GR × HL 75:25, which exhibited 23.3% higher mortality when exposed to 5000 ppm formalin (Figure 2).

**Figure 3:**
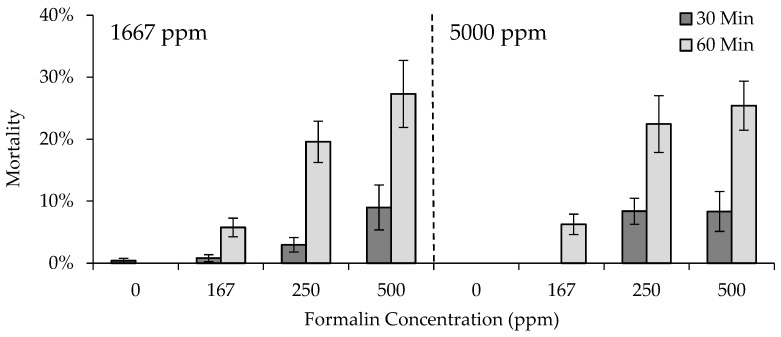
Average mortality (2SE bars) of rainbow trout (*Oncorhynchus mykiss*) exposed to either 1667 or 5000 ppm formalin as eggs (separated by vertical dotted line), and re-exposed to formalin as fingerlings at concentrations of 0, 167, 250, or 500 ppm for 30 or 60 min. Note that the mortality axis is reduced, to show differences among formalin concentrations and durations of exposure.

Fingerling mortality was affected by an interaction between egg formalin concentration, fingerling formalin concentration, and exposure duration, and differed by strain (Table 6). On average, mortality was higher in the GR strain (12.8 ± 4.4%) and GR × HL 50:50 (12.4 ± 4.8%) than the HL strain (3.8 ± 1.5%) or GR × HL 75:25 (5.1 ± 2.4%). Mortality increased as formalin concentration increased, and mortality within a concentration was higher, with a 60 vs. 30min exposure duration (Figure 3). In most cases, fingerling mortality did not differ as a result of egg formalin concentration. For example, fingerling mortality was similar for fish exposed to 500 ppm formalin for 60 min, despite previous exposure to either 1667 or 5000 ppm formalin as eggs. Increased mortality due to previous exposure to a higher formalin concentration as eggs (5.4%) was only observed in fingerlings exposed to 250 ppm for 30 min, but mortality did not exceed that of fish exposed to a higher formalin concentration or for a longer exposure duration (Figure 3).

### 3.3. Experiment 3: Fish Size, Feeding, Crowding, Flow, and Density

**Figure 4:**
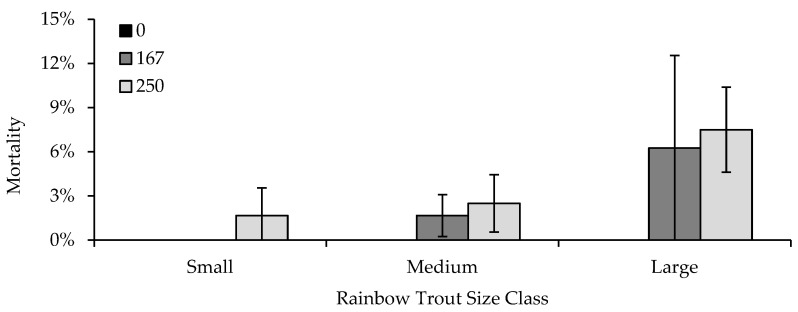
Average mortality (2SE bars) of small (38 mm total length [TL]), medium (77 mm TL) and large (128 mm TL) rainbow trout (*Oncorhynchus mykiss*) exposed to formalin concentrations of 0, 167, and 250 ppm. No mortality occurred when fish were exposed to 0 ppm (black bars) in any of the three size classes. Note that the mortality axis is reduced, to show differences among formalin concentrations and size.

With regard to fish size-at-exposure, an interaction between formalin concentration and fish size was the best predictor of mortality, although the additive model containing both factors also explained some of the variability in mortality (Table 7). Small fish (38 mm TL) were the least sensitive to formalin, with mortality occurring only in tanks where fish were exposed to 250 ppm formalin (Figure 4). Fingerling (medium; 77 mm TL) mortality occurred when fish were exposed to both 167 and 250 ppm formalin, but did not differ between the two concentrations or from that exhibited by small fish. Mortality increased with an increase in size, with higher mortality observed in large fish (128 mm TL) exposed to 250 ppm formalin, relative to small and medium fish. However, there was not a difference in mortality within large fish exposed to either 167 or 250 ppm formalin (Figure 4).

Feeding fish the day of treatment interacted with formalin concentration to affect fingerling rainbow trout mortality (Table 7). On average, mortality was lower in the tanks where fish had last been fed the day prior to exposure to formalin (2.1 ± 0.8%) compared to those fed on the day of formalin exposure (9.2 ± 4.2%). Feeding caused mortality to increase by 5.4% in fish exposed to 167 ppm formalin and by 8.8% in fish exposed to 250 ppm formalin. Mortality also differed by strain (Table 7), with the GR strain (1.7 ± 1.6%) and GR × HL 75:25 (1.7 ± 1.4%) exhibiting lower mortality than either the HL strain (5.6 ± 3.7%) or GR × HL 50:50 (6.1 ± 5.0%).

Formalin concentration had the largest effect on mortality in the trial where fish were crowded down away from the inflow (Table 7). Mortality increased with an increase in formalin concentration, with no mortality occurring when fish were exposed to 0 ppm formalin, 2.0 ± 1.3% mortality when exposed to 167 ppm formalin, and 5.8 ± 3.4% mortality when exposed to 250 ppm formalin. Crowding had a lesser effect on mortality (Table 7), but higher mortality was observed in the tanks in which fish had been crowded down (3.9 ± 2.5%) compared to those in which they had not been crowded down (1.4 ± 0.9%).

In the density and flow trials, an interaction between formalin concentration and flow had the largest effect on mortality, with density having a lesser effect (Table 7). Mortality differed by both formalin concentration and flow rate. In the tanks with decreased flow (3.8 Lpm), mortality for fish exposed to 167 and 250 ppm formalin was 4.5 ± 1.8% and 9.8 ± 2.8%, respectively, and was more than two times higher than in tanks with a normal flow rate (7.6 Lpm; 167 ppm: 1.1 ± 1.0%; 250 ppm: 4.0 ± 1.9%). Although the modeling results suggested a possible effect of density on mortality, mortality in the tanks with increased density (160 fish in total; 3.4 ± 1.2%) did not differ from mortality in tanks containing a normal density (80 fish in total; 3.0 ± 1.3%), despite the increase in density index from 0.16 to 0.32.

### 3.4. Experiment 4: Density and Multiple Exposures

**Figure 5:**
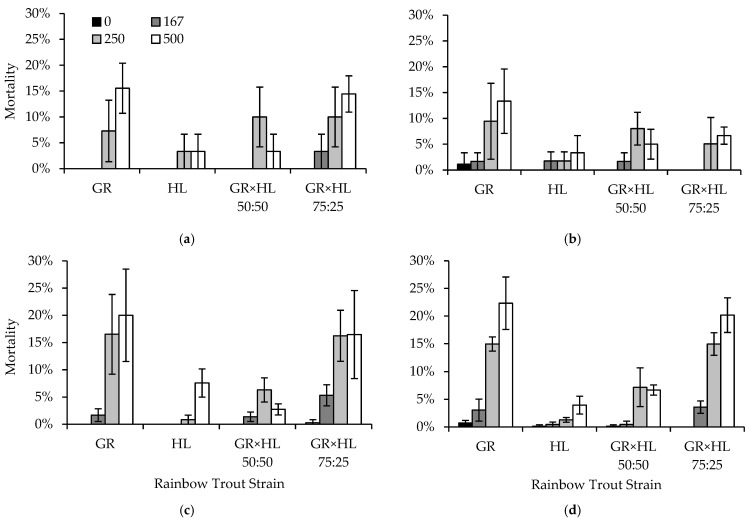
Average mortality (2SE bars) by strain for rainbow trout (Oncorhynchus mykiss) exposed to formalin concentrations of 0, 167, 250, and 500 ppm and reared at four densities: (**a**) 40 fish in total (density index [DI] = 0.09), (**b**) 80 fish in total (DI = 0.10), (**c**) 160 fish in total (DI = 0.30), and (**d**) 320 fish in total (DI = 0.49). Note that the mortality axes are reduced, to show differences among strains and formalin concentrations.

Density had a larger influence in experiment four, interacting with formalin concentration and strain to affect mortality of fingerling rainbow trout (Table 8). Mortality in the HL strain was generally lower than 5%, and the HL strain exhibited a sensitivity to formalin only in those tanks with a density index of ≥0.30. Neither the HL strain nor the GR × HL 50:50 exhibited an increase in mortality with an increase in tank density. The GR × HL 50:50 exhibited sensitivity to formalin when concentrations exceeded 250 ppm across all four densities; however, mortality was similar at 250 and 500 ppm formalin (Figure 5). The GR strain and GR × HL 75:25 exhibited sensitivity to formalin at all rearing densities, with higher mortality occurring at formalin concentrations of 250 and 500 ppm compared to 0 or 167 ppm. Additionally, especially for fish exposed to 250 or 500 ppm formalin, mortality for the GR and GR × HL 75:25 increased with an increase in tank density (Figure 5).

Treatment number (trend) appeared additively in the top model with the interaction between density, formalin concentration, and strain (Table 8); however, the small ΔAIC*_c_* value associated with the addition of a trend effect suggests that repeated exposure to formalin did not affect mortality, which was supported by the data. Mortality after the first exposure was 4.6 ± 1.0%, with an approximately half a percent increase in total mortality following subsequent exposures (exposure 2: 5.0 ± 1.1%; exposure 3: 5.6 ± 1.2%), but the cumulative effect of multiple treatments on mortality was small overall.

## 4. Discussion

Sensitivity to formalin, an increase in mortality with an increase in formalin concentration, was observed in both the egg and fingerling life stages of rainbow trout. Within the egg life stage, sensitivity was not observed for smaller increases in formalin concentration from 1667 to 2000 ppm, but higher mortality was observed when eggs were exposed to 5000 ppm formalin. Similarly, increased formalin concentrations of 250 and 500 ppm resulted in higher mortality in rainbow trout fingerlings, and mortality was affected by fish size and rearing conditions including flow, density, and feeding on the day of treatment. Sensitivity to formalin also differed by strain in both the egg and fingerling life stages.

A concentration of 1667 ppm formalin has been found to be effective for egg fungal control in a variety of salmonid species including rainbow trout [29,30], lake trout (*Salvelinus namaycush*) [33], landlocked fall Chinook salmon (*O. tshawytscha*) [34], and brown trout (*Salmo trutta*) [35]. Formalin concentrations up to 2000 ppm have been used in areas where higher fungal infection rates occur [8], and been shown to reduce bacterial abundance on the egg surface [6]. Our results show that increasing the formalin concentration from 1667 to 2000 ppm did not affect rainbow trout egg mortality, and that a concentration of 2000 ppm can be used if increased fungal or bacterial control is desired. Marking et al. [3] did not observe toxicity to rainbow trout eggs at a concentration of 5000 ppm formalin for exposures of 15 or 30 min. However, in our study the GR × HL 50:50 (experiment one) and GR × HL 75:25 (experiments one and two) exhibited higher egg mortality when exposed to 5000 ppm formalin, suggesting that this concentration is toxic to at least some strains of rainbow trout. Therefore, we recommend that formalin concentrations not exceed 2000 ppm unless higher concentrations have been tested and the potential toxicity is known. This recommendation is similar to and complies with the approved label claims for formalin which were based, in part, on previous evaluations of target animal safety. In these experiments, we concluded treatment once the eggs had eyed. All strains exhibited relatively low post-hatch mortality, and observation of mortalities in the tanks where fish were hatched indicated that fungal growth did not play a role in post-hatch mortality. However, others have suggested that if fungal growth contributes to post-hatch mortality, continuing formalin treatment through the eyed egg and sac fry stages may increase post-hatch survival [36].

Mortality for eggs treated with 1667 and 2000 ppm in our study were comparable to those observed with 1667 ppm in previous studies [29,30]. Strains differed in their mortality across concentrations, which has also been previously observed between the Cleghorn and Erwin rainbow trout strains [29]. In both experiments, the GR strain exhibited lower total mortality than the HL strain, which may be explained by the history of domestication of the GR strain [25] compared to the wild-origin HL strain [26]. The GR strain has been reared in hatcheries for over a century, likely being exposed to formalin through each generation produced, which has potentially reduced its sensitivity relative to its wild counterpart. The GR × HL 50:50 may have exhibited lower mortality as a result of heterosis. Similar effects have been observed with regard to increased tolerance of the GR × HL 50:50 to low dissolved-oxygen concentrations over both the GR and HL strains [24]. Additionally, the GR × HL 50:50, and other 50:50 crosses between the GR and wild-strain fish, have shown increased resistance to *M. cerebralis* over the wild strain [27,37,38,39], as well as advantages in swimming performance and post-stocking survival over the GR strain [40,41]. Outcrossing and backcrossing have been shown to affect survival following exposure to *M. cerebralis* [38], and may have similarly played a role in the higher egg mortality observed in the GR × HL 75:25, despite being higher proportion GR relative to the GR × HL 50:50. However, mortality results across the two experiments, especially in the GR × HL 50:50, suggest that mortality differences both within and among strains could be a result of differences in egg quality, which has also affected the results of other formalin exposure experiments [29,30,35].

Although formalin sensitivity has been evaluated in rainbow trout egg, sac fry, and fingerling life stages independently, to our knowledge, this is the first study to evaluate whether exposure to higher formalin concentrations as eggs resulted in increased sensitivity to formalin in the fingerling life stage. In most cases, fingerling mortality did not differ as a result of egg formalin concentration. Our results suggest that, in general, previous exposure to formalin did not affect mortality when exposed again at an older life stage, and there is evidence from some fish species that acclimation to lethal concentrations may increase formalin tolerance [7]. It is also possible that individuals that are susceptible to formalin will be so regardless of life stage, such that susceptible individuals died when exposed to formalin as eggs, reducing further mortality in the fingerling life stage. These results are supported by the lack of increase in mortality, despite multiple exposures during the fingerling life stage seen in experiment four.

Life stage [42] and fish size [43] have previously been shown to affect sensitivity to formalin. Similar to what we observed, Taylor and Glenn [43] showed that formalin generally appeared to be more toxic to larger rather than smaller rainbow trout, Chinook salmon, and Coho salmon *(O. kisutch*), which may be related to fish having a larger surface area capable of absorption [44], or a higher metabolic rate [11,44]. This can be an important consideration for not only the size or life stage of fish being treated, but also strain differences in growth or size-at-age. For example, the GR strain grows significantly faster than wild strains or their crosses, attaining weights two to three times that of the wild strain and 50:50 crosses of the same age [40]. Although we did not observe a strain effect in this portion of experiment three, growth or metabolic rate could account for strain mortality differences observed in other trials or in experiment four, as well as hatchery observations that GR-strain fish appeared to be more sensitive than other strains being treated on the same unit.

Pretreatment starvation of fish and maintaining optimal water quality conditions have been suggested as a means of controlling mortality during and following formalin exposure [11], and we tested the effects of flow, fish density, crowding fish away from the inflow of formalin, and feeding on the day of treatment, and multiple exposures on mortality in experiments three and four. Feeding fish on the day they were treated caused mortality to increase by up to 8.8% compared to fish fed the day prior to treatment. Ingestion of food increases oxygen consumption and elevates the metabolic rate [45]. Exposure to formalin may also induce an increased metabolic rate and oxygen demand [7]. The combined effects of feeding and formalin exposure may affect the ability of fish to maintain normal metabolism during and following treatment [14], and this, along with the potential for respiratory compromise during treatment [46], likely resulted in the increased mortality observed in the fed fish during our experiment. In addition, metabolic waste product concentrations increase following feeding [45], which, along with the presence of unconsumed feed, could decrease water quality and increase mortality during treatment. Therefore, our results support the fact that water quality must be optimal prior to initiation of treatment [7], and that pretreatment starvation, higher available oxygen levels, and lower metabolite levels will result in reduced mortality [11].

Contrary to the expectation that crowding fish down would decrease mortality by moving fish out of formalin hot spots located near the inflow, mortality was higher in tanks in which fish had been crowded down than in tanks where they had not been. Mortality in the crowded tanks may more closely reflect what would be expected when formalin is more evenly distributed, compared to when fish are congregated at the inflow and potentially able to avoid formalin that has not diffused throughout the water column. Alternatively, this increase in mortality may be a result of a relative increase in density in the tanks where fish were crowded away from the inflow, which increased the density index from 0.16 to 0.43.

Hatchery managers rear salmonids at various densities to meet production and stocking goals (reviewed by [47]). Increasing fish density provides a means to increase production without a concomitant increase in system costs [47]. However, fish reared at high densities have been shown to have lower dominance status, variable weight, length, and condition, and increased stress, all of which can lead to increased susceptibility to pathogen infection [48,49]. Rearing fish at higher densities can also lead to abrasions and reduced fin condition [50,51,52,53], and increased mortality [53,54], all of which can facilitate pathogen transmission and increase infection rates, and thereby the need to treat fish with formalin. Our results show that formalin treatment can increase mortality at higher rearing densities, especially in certain strains of rainbow trout. It is important to note that our experiments were conducted under ideal conditions with uninfected fish, and that our rearing densities, even in the high-density treatments, never exceeded the recommended density index of 0.5 [8]. Therefore, it is likely that mortality rates during treatment would be higher than those observed in our experiments if fish are both infected and reared at densities above this threshold. Lastly, density can also affect the efficacy of formalin treatments. For example, in steelhead rearing ponds, parasites cannot be controlled by formalin treatments if the rearing densities exceed 0.84 to 0.96 kg of fish per liter of water per minute at 15.6–21.1 °C [8]. Rearing fish at lower densities should not only reduce the transmission and incidence of disease, and thereby the necessity for formalin treatments, but also increase the efficacy of treatment and reduce mortality associated with treating infected fish with formalin.

Repeated exposure to formalin did not greatly affect mortality. However, mortality is not the only concern with regard to treating fish multiple times with formalin. In rainbow trout, exposure to formalin has been shown to cause hemorrhaging, hypertrophy, epithelial damage, and necrosis in the gill lamellae [14,15,16,17]; shrinkage and cytoplasmic degeneration of liver cells [18], and dilation, hemorrhaging, and damage of the liver blood vessels [16]; and a reduction of lymphoid tissue in the spleen [14]. Changes in water quality and use of higher formalin concentrations may exacerbate these effects or induce other histopathological changes in exposed fish. However, not all studies have shown negative effects of formalin exposure, even after multiple exposures. For example, repeated exposure to formalin caused only a slight non-significant increase in frequency of laminar fusion and number of lamellar mucous cells in Atlantic salmon (*S. salar*), and an increase in the number of mucous cells present on gill lamellae of rainbow trout [12]. In addition, Speare and MacNair [13] showed that growth rates, appetite, feed conversion, and body condition index of rainbow trout were not significantly affected by twice-weekly treatments with 200 ppm formalin in a 1 h static bath, and that fin condition and corneal opacity were better in treated versus untreated fish. Therefore, the decision to treat multiple times should be based on both the need for multiple treatments to help clear an infection, as well as knowledge of how the target population will respond to multiple formalin exposures.

The rainbow trout in our experiments exhibited differential mortality both across and within strains, depending on the rearing conditions or formalin concentrations to which they were exposed. Although various parameters of water chemistry and the physical conditions of the fish may influence formalin sensitivity in rainbow trout, genetics seem to be the major factor affecting mortality during formalin exposure [55], with differences in mortality of up to 60% between some rainbow trout strains [11]. Strains of brown trout have also exhibited differential sensitivity to formalin during treatment [56]. Formalin tolerance is a strongly heritable trait in rainbow trout, and successful breeding for formalin tolerance can be accomplished within relatively few generations [57]. In these fingerling experiments, contrary to our expectations, the GR strain was generally less sensitive to formalin exposure, perhaps a result of their history of domestication and heritability of tolerance, given regular exposure to formalin in both the egg and fingerling life stages. Treatment conditions also affected strain formalin sensitivity. Specifically, the GR-strain fish were less sensitive to formalin when fed on the day of treatment, potentially a function of their history of domestication, but these same fish exhibited higher mortality when tank density increased. Although genetics and history of domestication appear to play a role in formalin sensitivity, our results show that the conditions during treatment are also an important consideration for strain-specific mortality.

## 5. Conclusions

Our results show that there are differences in mortality between rainbow trout strains when exposed to formalin as eggs, and that the majority of the mortality occurs pre- versus post-hatch. In general, there were few differences in mortality within a strain at lower concentrations, but differences and mortality increased when concentrations of 5000 ppm were used. Therefore, we recommend not exceeding 2000 ppm formalin during the egg life stage. If fungal growth requires higher concentrations of formalin to be used, these higher concentrations should be tested on a smaller batch of eggs to determine their effect on mortality, or other means of fungal removal, such as water filtration, may be needed. Prior exposure to higher concentrations of formalin in the egg life stage did not appear to affect mortality when fish were exposed again at an older life stage. However, other factors may affect mortality in older life stages. In addition to the factors included in our experiments, water hardness and pH, which were not measured, have also been shown to affect formalin sensitivity in rainbow trout, and these, along with other water quality parameters, may be important to check, prior to initiating treatment. The fingerling mortality results presented herein should be considered minimum losses, because these experiments were conducted with healthy fish (i.e., no disease outbreaks), and losses are expected to be higher when fish are additionally stressed by the presence of external parasites. In conclusion, it will be important for hatchery managers to consider all factors prior to treating rainbow trout with formalin, including severity of disease outbreak, expected minimum losses, strain and life stage being treated, and hatchery conditions, including, but not limited to, type of culture (e.g., tank or raceway), flow, recent feeding, temperature, rearing densities, cleanliness, and water quality parameters.

## Figures and Tables

**Table 1:** Number of rainbow trout (*Oncorhynchus mykiss*) spawning pairs pooled to create eggs for the GR, HL, GR × HL 50:50, and GR × HL 75:25 strains for each of the four experiments (Exp 1–4). Two-year-old (2 yo) and three-year-old (3 yo) male (♂) and female (♀) fish were used to spawn each stain, dependent upon brood stock availability within these age classes in a given year. A “—” indicates that spawning pairs of an age and sex combination were not available for a given experiment.

Strain	Age and Sex	Exp 1	Exp 2	Exp 3	Exp 4
GR	2 yo ♀ × 3 yo ♂	13	18	14	21
HL	2 yo ♀ × 3 yo ♂	2	18	—	—
	3 yo ♀ × 2 yo ♂	6	3	12	12
GR × HL 50:50	2 yo GR ♂ × 2 yo HL ♀	—	20	—	—
	2 yo GR ♂ × 3 yo HL ♀	—	—	—	12
	3 yo GR ♀ × 2 yo HL ♂	5	—	—	—
	3 yo GR ♀ × 3 yo HL ♂	—	—	12	—
GR × HL 75:25	2 yo GR × HL 50:50 ♀ × 2 yo GR ♂	—	37	—	—
	2 yo GR × HL 50:50 ♀ × 3 yo GR ♂	6	—	—	—
	2 yo GR × HL 50:50 ♂ × 3 yo GR ♀	—	—	12	18

**Table 2:** Assignment of rainbow trout (*Oncorhynchus mykiss*) strain to replicate egg cups, numbered (#) 1 through 8 and assigned using a random number generator, within the top four of seven trays of the three Heath stacks used in Experiment 1, to which one of the formalin concentrations of 1667, 2000, or 5000 ppm was also assigned.

Tray #	Egg Cup #	Stack 1: 1667 ppm	Stack 2: 2000 ppm	Stack 3: 5000 ppm
1	1	GR × HL 75:25	GR	GR × HL 50:50
1	2	GR	GR × HL 75:25	GR × HL 75:25
2	3	GR × HL 50:50	HL	GR
2	4	GR	GR × HL 50:50	GR × HL 50:50
3	5	HL	GR × HL 75:25	GR
3	6	GR × HL 50:50	GR	HL
4	7	HL	HL	HL
4	8	GR × HL 75:25	GR × HL 50:50	GR × HL 75:25

**Table 3:** Assignment of rainbow trout (*Oncorhynchus mykiss*) strain to replicate egg cups, numbered (#) 1 through 12 and assigned using a random number generator, within the top six of seven trays of the two Heath stacks used in Experiment 2, to which one of the formalin concentrations of 1667 or 5000 ppm was also assigned.

Tray #	Egg Cup #	Stack 1: 1667 ppm	Stack 2: 5000 ppm
1	1	GR × HL 50:50	GR × HL 75:25
1	2	GR × HL 75:25	HL
2	3	GR × HL 50:50	GR × HL 50:50
2	4	GR	HL
3	5	GR	GR
3	6	HL	GR × HL 50:50
4	7	GR × HL 75:25	GR × HL 75:25
4	8	GR	GR × HL 50:50
5	9	HL	GR × HL 75:25
5	10	GR × HL 75:25	GR
6	11	HL	HL
6	12	GR × HL 50:50	GR

**Table 4:** Random assignment of formalin concentration (0, 167, 250, and 500 ppm) to the 74.8 L tanks, numbered (#) 1 through 12, used in each of the four trials conducted in Experiment 2 (trial 1: 30 min treatment of rainbow trout (*Oncorhynchus mykiss*) fingerlings previously exposed to 1667 ppm as eggs; trial 2: 60 min treatment of fingerlings previously exposed to 1667 ppm as eggs; trial 3: 30 min treatment of fingerlings previously exposed to 5000 ppm as eggs; trial 4: 60 min treatment of fingerlings previously exposed to 5000 ppm as eggs). Each tank contained 80 fish, 20 fish of each strain uniquely marked with a different color of VIE tag, to quantify strain mortality within a tank. A similar process was used to randomly assign formalin concentration to tanks in the trials conducted in Experiments 3 and 4.

Tank #	Trial 1	Trial 2	Trial 3	Trial 4
1	250	0	250	250
2	167	500	0	167
3	250	500	167	250
4	500	250	0	500
5	0	0	250	0
6	250	167	0	500
7	167	250	167	167
8	500	167	500	0
9	0	500	167	167
10	0	250	500	0
11	500	167	250	500
12	167	0	500	250

**Table 5:** Model selection results for factors affecting pre-hatch, post-hatch, and total mortality for rainbow trout (*Oncorhynchus mykiss*) eggs exposed to formalin concentrations of 1667, 2000, and 5000 ppm in Experiment 1. The candidate model sets each contained five models with singular, additive, and interactive effects of strain and formalin concentration on mortality; however, only models for which AIC*_c_* were nonzero (*w_i_* > 0) are shown. Models were ranked within each set by ΔAIC*_c_*, the AIC*_c_* value of the model relative to the best model in the set, and Akaike weights (*w_i_*), representing the probability that the model was the best model, given the data and the model set.

Mortality	Model	R^2^	log(*L*)	*K*	AIC*_c_*	ΔAIC*_c_*	*w_i_*
Pre-hatch	Concentration × Strain	0.98	97.86	14	−121.06	0.00	>0.99
	Strain	0.50	59.02	4	−107.93	13.13	<0.01
	Concentration + Strain	0.67	64.08	7	−107.17	13.89	<0.01
	Intercept	0.00	50.62	1	−99.06	22.00	<0.01
	Concentration	0.17	52.88	3	−98.55	22.51	<0.01
Post-hatch	Strain	0.87	83.37	4	−156.64	0.00	0.94
	Concentration + Strain	0.89	86.03	7	−151.05	5.58	0.06
Total	Concentration × Strain	0.99	98.38	14	−122.09	0.00	>0.99
	Strain	0.61	57.53	4	−104.96	17.12	<0.01
	Concentration + Strain	0.75	62.80	7	−104.59	17.49	<0.01

**Table 6:** Model selection results for factors affecting pre-hatch, post-hatch, and total mortality for rainbow trout (*Oncorhynchus mykiss*) eggs exposed to formalin concentrations of 1667, 2000, and 5000 ppm, and fingerlings exposed to formalin concentrations of 0, 167, 250, or 500 ppm for exposure durations of 30 or 60 min in Experiment 2. Egg-candidate model sets each contained five models with singular, additive, and interactive effects of strain and formalin concentration on mortality. The fingerling-mortality-candidate model set contained 22 models with singular, additive, and interactive effects of egg formalin concentration (Egg), fingerling formalin concentration (Fingerling), exposure duration (Duration), and strain. Only models for which AIC*_c_* were nonzero (*w_i_* > 0) are shown. Models were ranked within each set by ΔAIC*_c_*, the AIC*_c_* value of the model relative to the best model in the set, and Akaike weights (*w_i_*), representing the probability that the model was the best model, given the data and the model set.

Mortality	Model	R^2^	log(*L*)	*K*	AIC*_c_*	ΔAIC*_c_*	*w_i_*
Pre-hatch	Strain	0.64	65.22	4	−120.35	0.00	0.43
	Concentration × Strain	0.88	78.55	10	−120.17	0.18	0.39
	Concentration + Strain	0.71	67.80	6	−118.65	1.69	0.18
	Intercept	0.00	52.76	1	−103.34	17.00	<0.01
	Concentration	0.07	53.61	2	−102.65	17.69	<0.01
Post-hatch	Strain	0.41	70.37	4	−130.64	0.00	0.84
	Intercept	0.00	64.07	1	−125.96	4.68	0.08
	Concentration + Strain	0.43	70.84	6	−124.73	5.91	0.04
	Concentration	0.02	64.34	2	−124.11	6.52	0.03
	Concentration × Strain	0.48	71.89	10	−106.85	23.78	<0.01
Total	Strain	0.56	61.48	4	−112.86	0.00	0.58
	Concentration + Strain	0.65	64.14	6	−111.35	1.51	0.27
	Concentration × Strain	0.84	73.53	10	−110.15	2.71	0.15
	Intercept	0.00	51.57	1	−100.96	11.90	<0.01
	Concentration	0.09	52.66	2	−100.76	12.10	<0.01
Fingerling	Egg × Fingerling × Duration × Strain	0.64	491.25	20	−937.58	0.00	1.00

**Table 7:** Model selection results for factors affecting rainbow trout (*Oncorhynchus mykiss*) fingerling mortality in Experiment 3. Each of the four model sets included an intercept-only model, as well as singular, additive, and interactive combinations of factors affecting mortality, including strain and formalin concentration (all model sets), fish size (model set one), feeding (model set two), crowding (model set three), and density and flow (model set four). Candidate model sets for fish size, feeding, and crowding contained 13 models per set, whereas the density-and-flow-candidate model set included 40 models. Only models for which AIC*_c_* were nonzero (*w_i_* > 0) are shown. Models were ranked within each set by ΔAIC*_c_*, the AIC*_c_* value of the model relative to the best model in the set, and Akaike weights (*w_i_*), representing the probability that the model was the best model, given the data and the model set.

Factor(s)	Model	R^2^	log(*L*)	*K*	AIC*_c_*	ΔAIC*_c_*	*w_i_*
Fish Size	Concentration × Size	0.44	391.61	9	−762.79	0.00	0.65
	Concentration + Size	0.35	387.33	6	−761.57	1.22	0.35
Feeding	Concentration × Feeding	0.42	300.31	6	−587.33	0.00	0.48
	Concentration × Feeding + Strain	0.51	304.80	10	−586.00	1.33	0.25
	Concentration + Feeding	0.36	298.05	5	−585.19	2.14	0.17
	Concentration + Feeding + Strain	0.45	302.00	9	−583.10	4.23	0.06
	Concentration + Feeding × Strain	0.51	304.23	11	−582.06	5.27	0.03
	Concentration	0.25	292.78	3	−579.21	8.11	0.01
Crowding	Concentration	0.22	300.45	3	−594.54	0.00	0.62
	Concentration + Crowding	0.24	301.19	5	−591.47	3.07	0.13
	Concentration + Strain	0.31	303.48	7	−591.21	3.33	0.12
	Concentration × Crowding	0.28	301.98	6	−590.66	3.88	0.09
	Concentration + Crowding + Strain	0.34	304.38	9	−587.85	6.69	0.02
	Concentration × Crowding + Strain	0.38	305.39	10	−587.17	7.37	0.02
Density and Flow	Concentration × Flow	0.48	676.61	6	−1340.61	0.00	0.78
	Concentration × Flow + Density	0.48	676.85	8	−1333.63	3.98	0.11
	Concentration × Flow + Strain	0.50	678.35	10	−1335.05	5.55	0.05
	Concentration + Flow	0.44	672.40	5	−1334.37	6.24	0.03
	Concentration × Density + Flow	0.46	674.22	8	−1331.38	9.23	0.01
	Concentration × Flow + Density + Strain	0.51	678.62	12	−1330.85	9.75	0.01

**Table 8:** Model selection results for factors affecting rainbow trout (*Oncorhynchus mykiss*) fingerling mortality in Experiment 4. The candidate model set contained 52 models with singular, additive, and interactive effects of strain, formalin concentration, density, and multiple exposures (trend) on mortality; however, only models for which AIC*_c_* were nonzero (*w_i_* > 0) are shown. Models were ranked within each set by ΔAIC*_c_*, the AIC*_c_* value of the model relative to the best model in the set, and Akaike weights (*w_i_*), representing the probability that the model was the best model, given the data and the model set.

Model	R^2^	log(*L*)	*K*	AIC*_c_*	ΔAIC*_c_*	*w_i_*
Concentration × Density × Strain + Trend	0.70	1347.76	67	−2543.62	0.00	0.51
Concentration × Density × Strain	0.70	1343.88	64	−2543.50	0.12	0.49
Concentration × Strain + Density	0.63	1284.74	20	−2527.96	15.66	<0.01
Concentration × Strain + Density + Trend	0.63	1287.90	23	−2527.80	15.82	<0.01

## Data Availability

The data used to support the findings of this study are available from the corresponding author upon request.
